# High Prevalence of Livestock-Associated Methicillin-Resistant *Staphylococcus aureus* in Hungarian Pig Farms and Genomic Evidence for the Spillover of the Pathogen to Humans

**DOI:** 10.1155/2023/5540019

**Published:** 2023-02-21

**Authors:** Ervin Albert, Rita Sipos, Vincent Perreten, Ákos Tóth, Erika Ungvári, Márton Papp, Ádám Dán, Imre Biksi

**Affiliations:** ^1^Department of Pathology, University of Veterinary Medicine Budapest, Budapest, Hungary; ^2^SCG Diagnostics Ltd., Délegyháza, Hungary; ^3^Biomi Ltd., Gödöllő, Hungary; ^4^Institute of Veterinary Bacteriology, Vetsuisse Faculty, University of Bern, Bern, Switzerland; ^5^Department of Bacteriology Mycology and Parasitology, National Public Health Centre, Budapest, Hungary; ^6^Centre for Bioinformatics, University of Veterinary Medicine Budapest, Budapest, Hungary

## Abstract

Livestock-Associated Methicillin-Resistant*Staphylococcus aureus* (LA-MRSA) strains of clonal complex (CC) 398 are widely disseminated in pigs and are considered emerging pathogens in human medicine. To investigate the prevalence, genetic characteristics, and zoonotic potential of the pathogen in pig production settings, dust samples were collected from 40 pig operations in Hungary, along with nasal swabs of attending veterinarians and other swine professionals (*n* = 27) in 2019. MRSA isolates were further characterized by performing whole-genome sequencing and susceptibility testing. The whole-genome sequences of 14 human-derived LA-MRSA clinical isolates from the same year were also included in the study. The proportion of positive farms was 83% (33/40), and 70% (19/27) of the swine professionals carried the pathogen. All but one MRSA strain belonged to CC398, including the human clinical isolates. The core genome multilocus sequence typing (cgMLST) analysis revealed clusters of closely-related isolates of both environmental and human origin with a pairwise allelic distance of ≤24, and both cgMLST and single nucleotide polymorphism (SNP) analyses suggest recent transmission events between the farm environment and humans. Four human clinical isolates harboured the immune-evasion gene cluster, of which one was considered to be closely related to farm isolates. Half of the swine-related strains showed decreased susceptibility to eight or more antimicrobials, and along with human isolates, they carried eight different types of multidrug-resistance genes, including *cfr*. The results showed a dramatic increase in the occurrence of LA-MRSA in the swine industry in Hungary, compared with the 2% prevalence reported by the European Food Safety Authority baseline study in 2008. The wide range of antimicrobial resistance of the strains, accompanied by the emergence of the pathogen in humans — both asymptomatic carriers and diseased — call for revision of the risk posed by LA-MRSA to the public health.

## 1. Introduction

After the first report of livestock-associated methicillin-resistant *Staphylococcus aureus* (LA-MRSA) from swine and swine farmers in 2005, a Europe-wide baseline study was conducted in 2008 to estimate the burden of the pathogen on the swine farming sector (European Food Safety Authority [1]). With the participation of more than 5,000 holdings from 26 countries, the prevalence of MRSA infection in breeding and production holdings was 14.0% and 26.9%, respectively. However, except for some severely affected countries, such as Germany, Spain, and Italy, there were much lower prevalence estimates in most member states. Hungary reported 3/141 positive production farms, while none of the 40 breeding holdings surveyed at that time were positive. In most countries, the dominant genetic lineage was clonal complex (CC) 398; other lineages were rarely identified. In the subsequent decade, similar investigations have been performed in countries with a previously low prevalence. The results have indicated rapid spread and increased genetic variability of the pathogen in only a few years [2, 3].

Pig farmers and attending veterinarians of farms are at higher risk of carrying LA-MRSA [4]. Surveys have estimated as high as 77%–86% prevalence among people working in MRSA-positive farms [5, 6]. The colonization seems to be transient in most cases [7], but data also suggest long-term persistence [8]. Therefore, it is not surprising that LA-MRSA CC398 has also entered the human health care system in some European countries, initially recognized as an occupational hazard of patients who work in animal husbandry [4]. However, recent results suggest the spread of LA-MRSA among people without livestock contact, which is a probable consequence of the recently discovered readaptation of the bacteria to the human host [9]. Besides the pathogen's zoonotic potential, MRSA infections are difficult to treat due to the frequent resistance to several antibiotic classes.

Since the EFSA baseline survey in 2008, no such further official estimation of MRSA in pig settings has been made in Hungary. However, an increased prevalence is expected based on the growing annual number of MRSA-positive swine samples that have been cultured at the Diagnostic Centre of Production Animal Diseases (University of Veterinary Medicine, Budapest, Hungary) since the early 2010s. Meanwhile, the proportion of suspected LA-MRSA CC398 strains isolated from human samples has risen in the past few years, as observed by the National Reference Laboratory for Antimicrobial resistant bacteria (AMR NRL), National Public Health Centre, Budapest, Hungary. Thus, the aim of this study was to investigate the prevalence, genetic variability, and resistance of LA-MRSA strains in the Hungarian swine sector and to assess their zoonotic potential by comparing them with those of human clinical origin.

## 2. Materials and Methods

### 2.1. Origin of Swine-Industry-Related Samples

One hundred of the total 292 large-scale breeding and production farms with more than 100 sows were randomly selected across the country and invited to participate in the survey. This sample size was determined to be able to estimate the proportion of MRSA-infected farms. The Epitools package (available at https://epitools.ausvet.com.au/oneproportion) was used for this calculation, with an estimated proportion (target prevalence) of 0.7 (70%), a desired precision of the estimate of 0.1 (10%), and a 95% confidence interval of the estimate. The calculation suggested the random selection of 64 farms to reach the goals of the sampling. The 100 figure was used instead to allow for loss of farms due to refusal to participate (meaning a 64% response rate). Finally, two randomly chosen fattening units were also invited to complete the survey. Hence, the number of investigated farms was 40.

Environmental dust samples were collected between May 2019 and December 2019 from five different production units on each farm, whenever possible, representing more age or production groups. Samplers were asked to rub a minimum 0.5 m^2^ area per unit with a 5 × 5 cm sterile dry cotton swab. Swabs were then pooled in a sterile plastic bag and delivered to the laboratory at ambient temperature (2–25°C) within 10 days. A questionnaire was supplied to inquire about the basic farm-related data, the primary source of the breeding population, regular animal movements between farms, and information on previous occurrences of MRSA. The completed questionnaire and a signed consent form were mandatory for further processing of the samples. Veterinarians and farm managers (henceforth: swine professionals) of the respective holdings could also provide a sample voluntarily. Human sampling was performed as described previously [10].

### 2.2. Isolation and Characterization of the MRSA Strains

#### 2.2.1. Culturing the Bacteria

Upon their arrival, environmental swabs were incubated overnight (16–20 h) at 37°C in 100 ml Mueller–Hinton broth supplemented with 6.5% (w/v) NaCl. Subsequently, about 10 µl of the enrichment suspension was spread simultaneously onto chromogenic agar plates for the selection of *S. aureus* (BD BBL CHROMagar *Staph aureus*) and MRSA (BD BBL CHROMagar MRSA II, Diagon Ltd, Hungary) and incubated at 35 ± 1°C for 24–48 h. One colony per sample, showing the characteristics described by the manufacturer, was chosen from the MRSA selective medium and subcultured on Columbia sheep blood agar plates under the same conditions. If more than one phenotypic variant of presumptive MRSA was present on the chromogenic agar plate, then one colony of each variant was chosen. Human nasal swabs were processed the same way. Colonies from the *S. aureus*-selective plates were treated as described above, only if the MRSA medium yielded no isolates. Pure cultures of the bacteria were stored at −80°C until further investigation.

#### 2.2.2. Molecular Investigation and Antibiotic Susceptibility Testing

Initial identification of presumptive *S. aureus* and MRSA isolates included a multiplex polymerase chain reaction (PCR) targeting the *spa* gene, a species-specific marker of *S. aureus*; the *mecA* and *mecC* genes, confirming methicillin resistance; and the *lukS-PV/lukF-PV* gene, the marker of the human-related Panton–Valentine leukocidin virulence factor [11]. Only MRSA strains were characterized further.

The minimal inhibitory concentrations (MIC) of 19 selected antibiotics were determined by microdilution in Mueller–Hinton broth using the Sensititre EUST plates (Thermo Fischer Scientific). When applicable, MIC values were interpreted using the European Committee on Antibiotic Susceptibility Testing [12] criteria. In the case of sulfamethoxazole, the Clinical and Laboratory Standards Institute [13] criteria were applied. Epidemiological cut-off values were used to determine the wild-type susceptibility to kanamycin, mupirocin, streptomycin, and tiamulin according to the methodology recommended by the European Union Reference Laboratory for Antimicrobial Resistance [14].

Fifty-seven isolates were selected for whole-genome sequencing (WGS). Whole-genome sequences were obtained from a NEBNext Ultra II directional DNA library with TruSeq adapters on an Illumina NovaSeq 6000 sequencing system (2 × 150-bp paired-end reads) at the NGS Platform, University of Bern, Switzerland. The resulting Illumina reads were transferred to BIOMI Ltd., Gödöllő, Hungary, for further bioinformatic investigation. Whole-genome multilocus sequence typing (MLST) analysis was performed on assembly free (AF) datasets using the BioNumerics software package version 8.0 (Applied Maths NV, Belgium), as described previously [15]. Then, distance matrices were generated by selecting the subset of 1861 core genome (cg) loci of each isolate. To visualize genetic relatedness, the unweighted pair group method with arithmetic mean (UPGMA) trees was constructed based on the cgMLST allele matrices. No resampling was performed during the tree construction, and branch lengths were calculated according to the average allelic differences of the isolates. The trees were annotated based on the iTOL 6.3 online platform [16]. Clusters of closely-related isolates were defined according to previous recommendations, using a 24 allelic difference as a cut-off value [17]. Recent transmission events were considered if the pairwise allelic difference was lower between the two strains than the estimated median annual variability within a *S. aureus* population (≤5) [18]. *Spa* typing and classical MLST of the seven housekeeping gene loci was performed within BioNumerics. *De novo* draft genome contigs were assembled using SPAdes in BioNumerics [19], and RASTtk was used for the annotation of selected genomes [20].

Resistance genes and mutations generating antibiotic resistance and the *mec*-carrying cassette chromosome (SCC*mec*) type were investigated by using the online ResFinder 4.1 [21] and SCCmecFinder 1.2 [22] tools with default settings (threshold of coverage: 60%, threshold of identity: 90%). The presence of virulence genes was assessed by using the sequence extraction tool in BioNumerics and confirmed by mapping the assembled genomes against the given virulence gene as reference in Geneious Prime 2022.1.1 (Biomatters Ltd., New Zealand) with default settings. Hits of 100% coverage and >98% nucleotide identity were considered to be valid results. The homology of the annotated draft genomes to plasmids known to carry the *cfr* resistance gene, pSCFS3 (AJ879565.1), and pSA737 (KC206006.1), was investigated in the same way. The genetic vicinity of the identified *cfr* gene was also investigated by using Geneious Prime.

Furthermore, 14 human-derived MRSA CC398 isolates from 2019 were involved in the study. MRSA isolates obtained from human clinical samples were routinely submitted to the AMR NRL of the National Public Health Centre for molecular typing between January and December 2019. Strains were selected based on their nontypeability (NT) by SmaI-pulsed-field gel electrophoresis (PFGE) and *spa* type related to CC398. WGS was performed in the sequencing facility of the National Public Health Centre on the MiSeq platform (Illumina) using 150-bppaired-end chemistry. Raw reads were then analyzed at BIOMI Ltd. as described previously. WGS data from this study are available in the Sequence Read Archive under the project numbers PRJNA901421 and PRJNA893357.

Whole-genome data of nine LA-MRSA strains from Denmark were included in the cgMLST analysis for comparative purposes. The strains represent the three dominant CC398 lineages—L1, L2, and L3—as identified by Sieber et al. [23]. To reinforce the cgMLST results in the case of closely related isolates, single nucleotide polymorphism (SNP) analysis was performed by using an in-house pipeline on all 79 strains. The pipeline is detailed in Supporting Information S1. A maximum likelihood tree was generated and then visualized and annotated with iTOL.

## 3. Results

### 3.1. Number of MRSA-Positive Farms and MRSA in Swine Professionals

**Figure 1:**
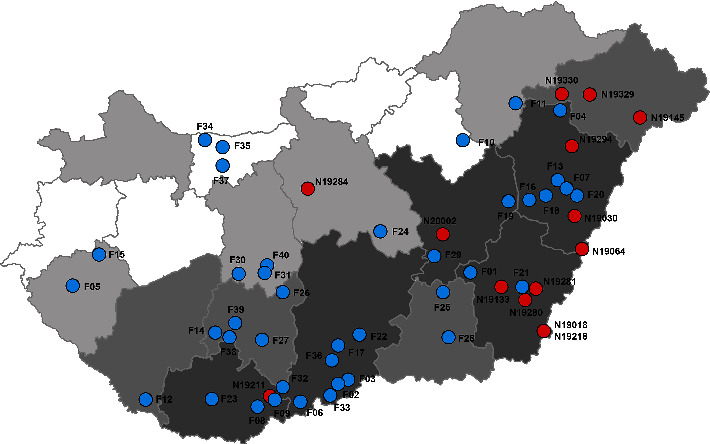
Location of farms and geographical origin of human clinical livestock-associated methicillin-resistant *Staphylococcus aureus* isolates involved in the study. Farms (F, blue circle) and places of origin of human clinical isolates (N, red circle) are numbered as referred to in the text. Counties are shaded according to the number of sows in the year 2019: 20,000–32,000 (dark grey); 10,000–20,000 (medium grey); 5,000–10,000 (light grey); and 1,000–5,000 (white).

A total of 40 holdings participated in the survey, including 38 farms rearing sows and two fattening farms, representing all major pig-producing regions of the country (Figure 1). The number of sows per breeding farm varied from 470 to 3000, with the total number accounting for 13% of the registered sows in Hungary in 2019 (National Food Chain Safety Office, Budapest, Hungary). Both fattening farms dealt with approximately 2000 fatteners per fattening cycle. Of the 40 sampled farms, 33 were MRSA positive (82.5%) during the recent sampling, and the proportion of the positive breeding and production farms was 31/38 (81.6%). Interestingly, only a quarter of the farms (10/40) reported a previous laboratory confirmation of MRSA. All but one previously MRSA-positive farm tested positive again. In the case of four farm samples, there were two distinct phenotypes present on the chromogenic agar plate-and both were selected for further testing-resulting in 37 farm isolates.

Among the 36 swine professionals in contact with the farms, 27 provided samples, of whom 25 were veterinarians and two were farm managers. Nineteen of them tested positive for MRSA (70.4%), including both farm managers. In a single case two phenotypically different isolates were further investigated from the same sample (Farm 28); thus, there were a total of 20 swine professional isolates. The number of MRSA-positivefarm-swine professional couples was 14. In only two cases, both the farm and the contact person were negative. In six cases the farms tested positive, while the swine professionals tested negative, and in five cases the farm was negative but the human sample tested positive.

Of note, none of the MRSA-negative samples yielded *S. aureus* on the *S. aureus*-selective chromogenic agar medium cultured in parallel, while other staphylococci grew on most plates, ruling out failure of sampling, or culturing in these cases (data not shown).

### 3.2. Initial Characterization of Farm-Related and Human Clinical MRSA Isolates

Among the 57 MRSA isolates chosen for further testing, all but a sequence type 45 (ST45) isolate belonged to MLST CC398, of which most represented the founder ST, ST398 (52/56). Three isolates were ST541, a single locus variant (SLV) of ST398, and a new SLV was also identified in the case of the isolate F19_E1, assigned to ST6268. An array of *spa* types related to CC398 were identified, with t034 (*n* = 22; 38.6%) and t011 (*n* = 21; 36.8%) being the two most prevalent variants. A few other isolates carried *spa* types t4208 (*n* = 3) and t1197 (*n* = 2), and one each represented *spa* types t571, t1250, t1255, t1451, and t4571. A new, yet unassigned *spa* type was identified among three further isolates by the WGS-based analysis, all originating from the same farm. The vast majority of the isolates carried the Vc (5C2&5) variant of the *Staphylococcus* cassette chromosome *mec* (*n* = 55), and a single isolate had type IVb(2B). A swine professional isolate was *scn* positive, carried SCC*mec* type IVa, identified as *spa* type t330, and belonged to a human-associated ST45. This isolate was considered not to be livestock‐associated and was excluded from subsequent analyses.

The human clinical isolates (*n* = 14) all belonged to CC398, and except for three isolates, they were all typed as ST398. Two isolates (N19145 and N19284) belonged to ST1232 and one isolate (N19018) was an SLV of ST398 and assigned to a new ST, ST8001. Six isolates were *spa* type t011, five were t034, two were t4208, and one was t3275. Except for two, all isolates belonged to the Vc (5C2&5) SCC*mec* type. The two ST1232 isolates lacked a second *ccrC1* allele and thus were typed as V (5C2). The major characteristics of the isolates that underwent WGS are summarized in Supporting Information S2.

### 3.3. Phenotypic Antimicrobial Susceptibility and Resistance Genes of Farm-Related Isolates

All analyzed farm-related LA-MRSA isolates (*n* = 56) were susceptible to rifampicin, vancomycin, mupirocin, and sulfamethoxazole, while resistance to fusidic acid or linezolid was observed only in single isolates (1.8%) (Table 1). In addition to resistance to the beta-lactam antibiotics penicillin and cefoxitin, all isolates were also resistant to tetracycline. It should be noted that three isolates were apparently susceptible to cefoxitin (MIC = 4 mg/L) but considered to be MRSA as these strains had previously grown on selective medium containing cefoxitin and tested positive for the *mecA* gene by PCR. There was higher resistance in the case of clindamycin (83.9%), trimethoprim (57.1%), and quinupristin/dalfopristin (48.2%). Almost three quarter of the isolates showed a non-wild-type phenotype when tested with tiamulin. Half of the isolates were resistant or expressed a non-wild-type phenotype to at least six tested antibiotics other than beta-lactams (Supporting Information S2).

Twenty-four resistance genes and three nonsynonymous point mutations in two genes conferring resistance to antibiotics were identified, in good agreement with phenotypic data (Table 2, Supporting Information S2). However, there were also discrepancies between the phenotypic and genotypic results in several isolates (Supporting Information S3). Eight isolates showed resistance towards chloramphenicol, without an identifiable underlying genetic trait. In addition, 14 isolates were apparently resistant to the quinupristin/dalfopristin combination (≥4 mg/L); however, each of them carried only the *lsa*(E) gene conferring resistance to streptogramin A antibiotics.

### 3.4. Relatedness of MRSA CC398 Isolates

The allelic differences among the 70 compared isolates ranged from 0 to 189.8 in almost all cases based on the cgMLST analysis (Supporting Information S4). There were two pairwise comparisons, however, where the differences were >200 and not displayed by the BioNumerics software. These were the human-derived clinical isolates of ST1232, which were distantly related to all others (minimum allelic difference >100) and thus were considered to be outliers (N19145 and N19284). On the other hand, they were rather closely related and showed a 5.0 allelic difference. The groups of farm environmental and swine professional isolates showed comparable yet moderate within-group heterogeneity, with allelic differences ranging from 0 to 125.9 (median 81.5) and from 0 to 123.0 (median 81.2), respectively. Except for the two outliers, the human clinical isolates were more distantly related, differing by a minimum of 29.3 alleles (maximum: 189.8, median: 144.6).

**Figure 2:**
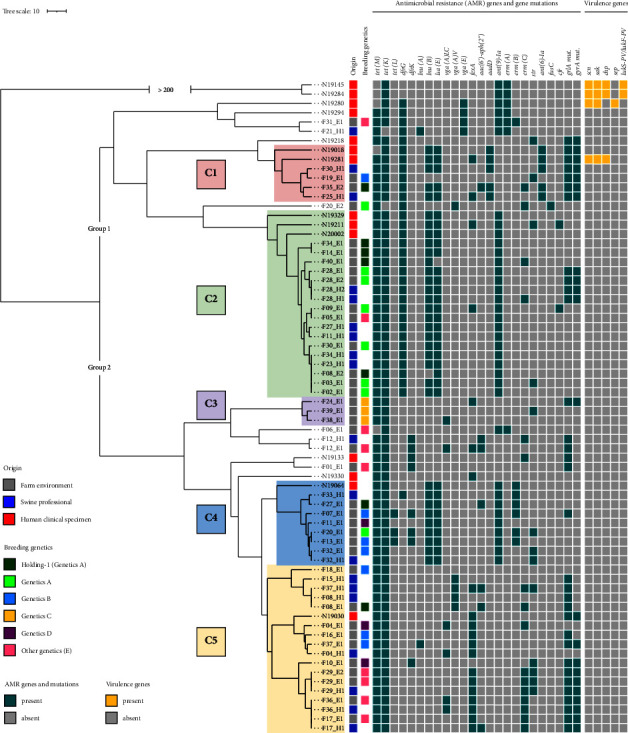
Relatedness and genetic traits of livestock-associated methicillin-resistant *Staphylococcus aureus* (MRSA) clonal complex (CC) 398 strains isolated from farm environments, swine professionals, and human clinical specimens. The unrooted unweighted pair group method with arithmetic mean (UPGMA) tree was generated using the core genome multilocus sequence typing (cgMLST) distance matrix of 70 MRSA isolates. The identified genetic clusters (C1–C5) are highlighted. The tree scale bar indicates a 10 allelic difference. The branch showing an allelic difference >200 was trimmed automatically by the BioNumerics software. Please note the strong association between the farm genetics and the clustering of isolates in C2 and C3.

Both UPGMA trees, with (Supporting Information S5) or without (Figure 2) the Danish isolates, were split into two main groups, Groups 1 and 2. The groups comprised clusters of closely-related isolates (Clusters C1 to C5), identified based on the trees and by applying the threshold of ≤24 allelic differences.

**Figure 3:**
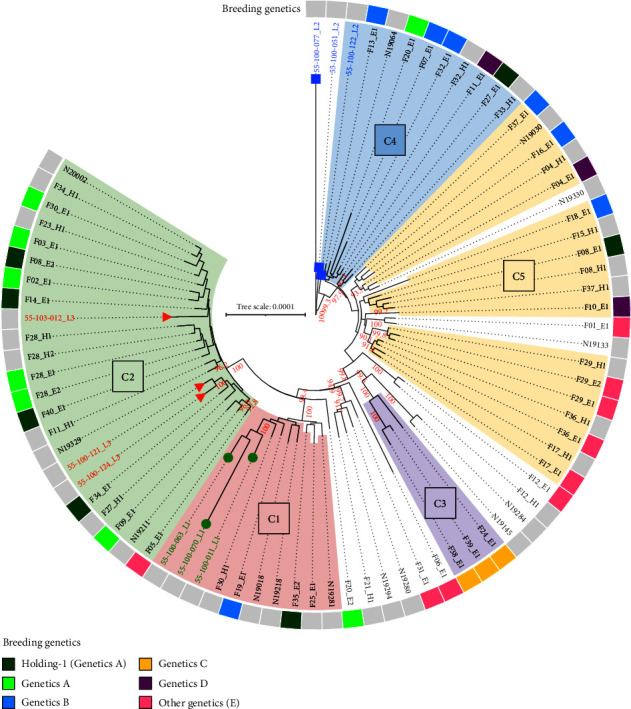
Maximum likelihood phylogeny of Hungarian and Danish livestock-associated methicillin-resistant *Staphylococcus aureus* (LA-MRSA) isolates. The colors indicate the clusters identified by core genome multilocus sequence typing (cgMLST) analysis. The scale bar indicates substitutions per site; bootstrap values are shown if >90 (red letters). There is a clear clustering of Danish L3 isolates (red triangle) with the Hungarian C2 cluster. The L1 isolates (green circle) are clustered with C1 isolates, but rather distantly-related. L2 isolates (blue square) are more distantly-related to the Hungarian LA-MRSA strains.

The SNP analysis identified 26–946 pairwise SNP differences (median: 241) in the case of all 79 LA-MRSA isolates. Within-group heterogeneity was indicated by 41–485 pairwise SNP differences (median: 239) among the farm environmental isolates, and the swine professional strains differed by 44–281 SNPs (median: 220). The human clinical isolates showed 37–335 SNP differences (median: 236) within the group. The maximum likelihood phylogeny tree reflected most characteristics of the UPGMA trees, as the previously identified clusters C1–C3 were well recognizable, while the genetic heterogeneity of C4 and C5 became more apparent (Figure 3).

### 3.5. Evaluating the Relatedness of Swine Professional, Farm Environmental, and Human Clinical Isolates

All whole genome–sequenced swine professional LA-MRSA isolates (*n* = 19) showed close relatedness (≤24.0 allelic difference) to at least one environmental isolate. When considering the 13 cases where both the farm and the swine professional tested positive for LA-MRSA, there were almost identical pairs. In eight cases, the allelic difference between the environmental and swine professional isolates was between 0 and 4.5, and the pairwise SNP difference was between 26 and 73. From the remaining five environments, three nasal swab isolate pairs showed 114.4–122.5 allelic differences or 230–248 SNPs. In one case (Farm 8), there were two phenotypically distinct environmental strains isolated, of which one did not differ from the nasal swab isolate (F08_E1; 0 allelic difference and 73 SNPs), while the other was rather distantly related (F08_E2; >100 allelic difference and >247 SNPs). All swine professionals who carried other genotypes than those detected on the farms were veterinarians. Only one of them reported no professional contact with other animals; the rest either worked with other farm animal species, mainly ruminants (*n* = 1), or also with swine on other farms (*n* = 2).

Nine out of 14 human clinical isolates clustered together with at least one farm environmental isolate, which was considered to be closely-related based on the cgMLST analysis (Figure 2). Two of them also showed <5 allelic differences with farm isolates (N19211 and N20002). Of note, isolates N19211 and F09_E1 (2 allelic differences or 52 SNPs) both exhibited the phenicol-lincosamide-oxazolidinone-pleuromutilin-streptogramin A (PhLOPS_A_) multiresistance pattern. The resistance could be attributed to the *cfr* gene, encoded on a large pSA737-like plasmid 40 kB in size.

Four isolates carried the immune evasion cluster (IEC) gene cluster, but only one of them showed a minimum of 15 pairwise allelic differences or ≥56 SNPs compared with farm environmental isolates; the rest were separated by a minimum of 48.4 allelic differences or 105 SNPs. Two of them were the ST1232 outliers, isolates N19145 and N19284. Only these two isolates encoded the Panton–Valentine leukocidin genes (*lukS-PV/lukF-PV*).

### 3.6. Farm Breeding Genetics and Relatedness of MRSA Isolates

**Figure 4:**
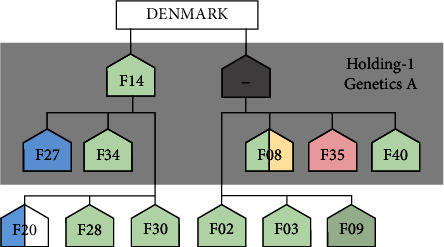
Overview of the purchase network of Holding-1 rearing breeding Genetics A. The farm shading corresponds to the Methicillin-Resistant *Staphylococcus aureus* clusters identified by core genome multilocus sequence typing (cgMLST) analysis: C1 (red), C2 (green), C4 (blue), C5 (yellow), and no identified genetic cluster (white).

In some cases, there was a strong association between the farms' breeding genetics and the genetic clustering of corresponding isolates. Farms could be classified into four well-defined groups (A–D) according to their breeders' genetics, while Genetics E comprised all other farms with mainly highly mixed breeds. Purchase networks of holdings were also identified. Holding-1 is a large integration with two nucleus farms, which has imported breeding Genetics A exclusively from Denmark in years prior to the recent study. These two nucleus farms, including Farm 14, supplied the multiplier and production farms of the integration (*n* = 5) and farms of other holdings (*n* = 6) (Figure 4). Most farms of Genetics A (10/12) clustered in Group 1, and mainly within cluster 2, according to the cgMLST analysis.

All but one farm breeding Genetics B were clustered in C4 (*n* = 3) and C5 (*n* = 3) (Figures 2 and 3). The farms of Genetics C had purchased their animals from the same source and formed the well-separated cluster C3 in Group 2. The three farms rearing Genetics D were scattered among C4 (*n* = 1) and C5 (*n* = 2), rather distantly-related (pairwise allelic difference >17.5). Among the farms of different mixed genetics, there were more in each main genetic group (Groups 1 and 2) of the cgMLST similarity trees. However, Farms 17, 29, and 36 formed a well-separated subcluster within C5 (pairwise allelic difference <3.1), and two of these farms were known to supply each other with animals (Figures 2 and 3).

### 3.7. Relatedness of Hungarian and Danish LA-MRSA Isolates

According to the cgMLST tree, the randomly selected Danish isolates of Lineages 1 and 3 clustered together with the Hungarian MRSA strains (Supporting Information S5). The three L1 isolates showed 10.1–27.2 allelic differences from the C1 strains, and similarly, L3 isolates showed 5.1–34.3 allelic differences from C2 isolates. Despite no obvious clustering, the L2 isolates were rather close to the Hungarian C4 strains, showing 8.3–45.5 (median: 17.5) pairwise allelic differences. The SNP analysis reinforced only the closely relatedness of all three L3 to C2 isolates, while only two L1 isolates and a single L2 isolate fell close to the C1 and C4 strains on the phylogenetic tree, respectively (Figure 3).

## 4. Discussion

Hungary used to be a country characterized by a low prevalence of MRSA according to a survey conducted by the EFSA in 2008 [1], as MRSA could be detected in none of the 40 breeding herds examined. Similarly, only 3 out of the 141 Hungarian production farms were positive (2.1%). The present study included a total of 40 pig farms in Hungary. The majority of these were production farms (*n* = 36), two were pig fattening operations, and only two farms corresponded to the “breeding farm” category as defined by the EFSA [1]. This is why it was not possible for us to conduct an analysis according to the categories used in the 2008 survey. As the composition of this sample is characterized by the predominance of commercial pig-producing farms, the indices of this latter category are also considered.

Compared with the data obtained in 2008, there had been a dramatic increase in prevalence: MRSA could be detected in 82% of the pig herds rearing sows. Based on a 10-year perspective, this trend could be expected on the basis of studies performed in other countries a few years after the European baseline survey. Between 2008 and 2012, the prevalence increased from 2.1%–3.4% to 23.6% in Poland and from 35.9%–40.0% to 65.5% in Belgium, and this was accompanied by an increase in the genetic variability of the identified strains [2, 3]. In the case of Poland, this phenomenon was explained by the increasing proportion of breeding pigs imported from countries characterized by a high prevalence of MRSA, including Germany and the Netherlands. In 2012, Denmark also belonged to that category, with an increase in the positive herds of 0.0%–3.5% to nearly 70% in 2014; however, in Denmark, the population structure of the strains seemingly became more uniform, as opposed to the examples cited above. Based on the genomic analysis of strains originating from that period and the retrospective study of animal transports, the pyramidal structure of the Danish pig industry and the one-way movement of animals within that structure proved to be the most important factors [23].

In Hungary, the imports of breeding pigs have continuously increased since the 2010s, and structural changes have occurred in the pig industry. These factors may have played a role in the wide dissemination of MRSA. Seven of the sampled 40 pig herds studied in Hungary belonged to an integration, Holding-1, which besides using imported breeding genetics of Danish origin (Genetics A) has applied the Danish pyramidal structure. The movement of animals was hierarchically organized within the holding, and at the time of the survey, it supplied a further six sampled farms with breeding animal replacements. Other herds had used two types of genetics of Dutch origin, but our sample also included farms working with hybrid genetics produced in Hungary (Farms 24, 38, and 39), as well as other herds using miscellaneous breeding genetics.

The low number of Hungarian isolates (*n* = 3) included in the 2008 EFSA survey does not allow us to draw conclusions regarding the genetic variability of the Hungarian MRSA population typical of that time. The genetic characteristics of the MRSA strains isolated in the current study, however, indicate the exclusive spread of strains belonging to the CC398 lineage, as seen in the other European countries [2, 24–26].

Comparison of the whole genomes also pointed to the important role played by the interherd trade of animals. The strains of clusters C1–C5 were grossly arranged according to the breeding genetics used in the herds. Clusters C1 and C2 mostly included the isolates obtained from Holding-1 working with Genetics A and from the farms supplied by it. The strains of the two clusters showed numerous similarities in both their core genomes and their resistance set encoded by their accessory genomes. Moreover, the inclusion of Danish LA-MRSA sequence data revealed a close phylogenetic relationship between more selected Danish L1 and L3 strains [23] and the Hungarian C1 and C2 isolates, respectively. In the light of these, it is reasonable to suppose that the conditions of the integration can be considered largely a Hungarian reflection of the Danish example, accompanied by the circulation of LA-MRSA strains of Danish import pig origin.

Researchers obtained similar results by comparing MRSA isolates from Southern Italian pig farms with genomic data originating from the previous Danish survey as well as from a study analyzing samples from several European countries [27]. Based on the analysis of the whole genomes, the strains isolated from pig farms rearing animals imported from Denmark could be assigned to one of the two dominant clusters (L1 and L3) of the Danish MRSA strains. In addition, the Danish and Italian strains also showed many similarities in their resistance profiles [26]. There were similar patterns in the case of the other smaller groups of Hungarian farms, further supporting the importance of animal movements between herds regarding the spread of MRSA.

Of note, there were some exceptions to the abovementioned trend. Based on their isolates, some farms working with Genetics A were also included in main genetic Group 2, and vice versa, MRSA strains belonging to C1 were isolated also from farms using other breeding genetics. Based on earlier experience, in rare cases the simultaneous presence of multiple types of CC398 clonal lines in a given pig farm also occurs [24, 28]. In the case of the samples evaluated in this study, due to the divergent phenotype of the bacterial colonies, their parallel testing seemed to be justified. However, from most of the samples only one bacterial colony was selected for further study, an approach that prevented the identification of variants that could be genetically very different but with similar phenotypes present in the culture. Such genetically different lines may be introduced to pig farms not only with carrier pigs but also with dust, by infected rodents or insects, and also by humans permanently colonized by them [29].

Working with livestock substantially increases the risk of colonization and developing a clinically apparent infection [30]. People working in pig operations are at an especially high risk [4]. Although humans seem to carry MRSA of animal origin only transiently [7], other studies conducted in pig farms indicate that permanent colonization is common among farm workers [8]. In the present study, two-thirds of the professionals working in pig farms, a total of 18 people, carried an LA-MRSA CC398 strain. In half of the cases the pig farm was clearly identifiable as the source of colonization, based on the high degree of similarity between the strains (0–4.5 cgMLST allelic difference and 26–72 SNPs). In the remaining cases, the farm was either negative or genetically different isolate was found. As at the time of the study, the veterinarians involved were also attending other pig farms or other livestock not included in the study, the human strains different from the farm strains or those isolated from farms with a negative status may have been originated from other sources [31]. Similarly, as mentioned before, in the current study the presence of multiple clonal lines not identified on the farms cannot be excluded.

The majority of human clinical samples included in this study are presumably of livestock origin. This seems to be supported by the fact that the isolates were mostly derived from the major pig-producing regions of Hungary. Although no further data of the patients supplying the samples, including their occupation, are known, a high animal density at the place of residence is a known risk factor for the human population that is not associated with animal production [32]. During the genetic comparison, these isolates were largely mixed with strains of pig origin, and in most cases, a direct spread of pig farm origin could be supposed based on the high degree of similarity found in the cgMLST and SNP distance matrices and resistance profiles. This view is consistent with the results of other studies, where the majority of LA-MRSA strains isolated from clinical cases may have been introduced into human health care directly from animal production, retaining their major characteristics [9, 24]. Except for two isolates, all of the clinical isolates in this study showed a high degree of resistance and also carried resistance genes against active compounds used almost exclusively in livestock, such as tiamulin and phenicols.

The host and ecological niche adaptation of MRSA CC398 is accompanied by the gain and loss of mobile genetic elements, like the human-associated IEC [9]. Although regaining the IEC seems not to be a prerequisite for the pathogen's survival or pathogenicity in the human host, its appearance in LA-MRSA strains is an indicator of the spillover to humans, according to the latest research [33]. Four of the strains in this study carried the IEC, but only one of them (N19281) showed a closer relationship with the strains of pig origin. Due to the low number of samples and the lack of metadata, no sound conclusions can be made regarding the microevolutionary changes of the Hungarian LA-MRSA population. However, the emergence of IEC-carrying LA-MRSA isolates call for further studies to assess the possibility of human-to-human spread and the risk posed by such a scenario to public health in Hungary.

Even if the matter of host adaptation is somewhat controversial, the high level of antibiotic resistance of LA-MRSA is already a public health concern, as half of the strains isolated from pig farms showed resistance or a non-wild-type phenotype to at least six tested active ingredients besides beta-lactams. Of the encoded resistance determinants, the multidrug resistance (MDR) genes that provide resistance to multiple antimicrobial agents cause the greatest concern. Almost all MDR genes of this study are known to be coded on mobile genetic elements and have been previously detected in staphylococci of both human and animal origin [34].

The plasmid-borne* cfr* gene identified in a pig farm isolate and in a human clinical isolate in this study deserves particular attention, as it encodes resistance to five classes of antimicrobials at the same time (the PhLOPS_A_ resistance pattern) [34]. Of the compounds involved, the oxazolidinone derivative linezolid is especially important in human medicine, as it is one of the antibiotics that can be used against MDR Gram-positive bacteria and primarily against MRSA [35]. The importance of linezolid resistance is underlined by the fact that MRSA, other *Staphylococcus* spp., and *Enterococcus* spp. strains carrying the *cfr* gene can cause nosocomial infections [36–38]. Despite its importance, however, it is reassuring that the data obtained in the years since the first detection of the *cfr* gene do not suggest an epidemic-like spread of this resistance gene [39, 40]. At the same time, pleuromutilins and florfenicol widely used in animal production exert high selection pressure for the enrichment of this resistance gene not only in the case of MRSA strains but also in other species. Therefore, the detection of this resistance gene in Hungary and the presence of *cfr*-positive MRSA calls attention again to the increased public health risk posed by the high-level MRSA carriage of people working in the pig industry [9].

The present study has some limitations. On the one hand, factors influencing the risk of MRSA occurrence on a pig farm are not completely known; hence, appropriate stratified random sampling was not possible. Although the sampled farms were randomly selected to eliminate some bias in sampling, the results were not intended to be representative for the Hungarian swine population. On the other hand, only 38 of the hundred selected farms took part in the study, less than the number needed for a proper estimate (64 farms). The low response rate further underlines that the present prevalence estimate might deviate from the (yet unknown) true prevalence of MRSA-infected Hungarian swine farms. It should be noted, however, that the results may adequately reflect the conditions of the important pig-producing regions of the country (Figure 1). In addition, when evaluating the obtained prevalence data, the fact that the method of sampling may reduce the sensitivity of the test must be considered. In a preliminary study, the EFSA found that the method based on testing dust samples, used to survey the infection status of pig farms, was somewhat less reliable than simultaneously sampling 60 pigs [41]. Therefore, when evaluating the results of the baseline survey conducted in 2008, the EFSA called attention to the fact that the actual prevalence was presumably higher than that suggested by the obtained data, even in areas showing an apparently low prevalence [1]. Taking the abovementioned facts into account, the number of infected farms in the present study may be lower than the actual number. This seems to be supported by the fact that pigs from some farms found to be negative during the study subsequently yielded MRSA strains in further routine diagnostic investigations (data not shown). Nonetheless, due to its easy and rapid implementation, the collection of environmental dust samples is a widespread method for the detection of MRSA in animal populations, in most cases supplemented by the collection of samples from animals [2, 42, 43].

In conclusion, the results of this study showed that livestock-associated MRSA has become extremely prevalent in Hungarian pig farming. In accordance with other findings from then-low prevalence countries, like Denmark or Poland, both massive live animal importing and interherd movement of positive animals could contribute to its rapid spread. Besides being abundant, most strains presented a wide range of antimicrobial resistance and the capacity to colonize and infect humans. Hence, the role of LA-MRSA as a zoonotic pathogen should also be re-evaluated and its epidemiology regularly monitored in Hungary.

## Figures and Tables

**Table 1:** Phenotypic antimicrobial susceptibility of 56 Swine-Related Livestock-Associated Methicillin-Resistant *Staphylococcu saureus* isolates.

		*Farm environment isolates (n* *=* *37)*			*Swine professional isolates (n* *=* *19)*			*Total (n* *=* *56)*		
		*R*(%)	*I*(%)	*S*(%)	*R*(%)	*I*(%)	*S*(%)	*R*(%)	*I*(%)	*S*(%)
Penicillin		37 (100)	—	0 (0)	19 (100)	—	0 (0)	56 (100)	—	0 (0)
Cefoxitin		36 (97.3)	—	1 (2.7)	17 (89.5)	—	2 (10.5)	53 (94.6)	—	3 (5.4)
Tetracycline		37 (100)	0 (0)	0 (0)	19 (100)	0 (0)	0 (0)	56 (100)	0 (0)	0 (0)
Clindamycin		29 (78.4)	0 (0)	8 (21.6)	18 (94.7)	1 (5.3)	0 (0)	47 (83.9)	1 (1.8)	8 (14.3)
Trimethoprim		21 (56.8)	—	16 (43.2)	11 (57.9)	—	8 (42.1)	32 (57.1)	—	24 (42.9)
Quinupristin/dalfopristin		20 (54.1)	4 (10.8)	13 (35.1)	7 (36.8)	8 (42.1)	4 (21.1)	27 (48.2)	12 (21.4)	17 (30.4)
Ciprofloxacin^†^		15 (40.5)	3 (8.1)	—	10 (52.6)	5 (26.3)	—	25 (44.6)	8 (14.3)	—
Chloramphenicol		17 (45.9)	0 (0)	20 (54.1)	7 (36.8)	0 (0)	12 (63.2)	24 (42.9)	0 (0)	32 (57.1)
Erythromycin		15 (40.5)	0 (0)	22 (59.5)	8 (42.1)	0 (0)	11 (57.9)	23 (41.1)	0 (0)	33 (58.9)
Gentamicin		4 (10.8)	—	33 (89.2)	3 (15.8)	—	16 (84.2)	7 (12.5)	—	49 (87.5)
Fusidic acid		1 (2.7)	—	36 (97.3)	0 (0)	—	19 (100)	1 (1.8)	—	55 (98.2)
Linezolid		1 (2.7)	—	36 (97.3)	0 (0)	—	19 (100)	1 (1.8)	—	55 (98.2)
Rifampicin		0 (0)	0 (0)	37 (100)	0 (0)	0 (0)	19 (100)	0 (0)	0 (0)	56 (100)
Vancomycin		0 (0)	—	37 (100)	0 (0)	—	19 (100)	0 (0)	—	56 (100)
Sulfamethoxazole^‡^		0 (0)	—	37 (100)	0 (0)	—	19 (100)	0 (0)	—	56 (100)

	ECOFF (mg/L)	NWT (%)		WT (%)	NWT (%)		WT (%)	NWT (%)		WT (%)
Tiamulin^§^	2	25 (67.6)		12 (32.4)	16 (84.2)		3 (15.8)	41 (73.2)		15 (26.8)
Streptomycin^§^	16	9 (24.3)		28 (75.7)	5 (26.3)		14 (73.7)	14 (25.0)		42 (75.0)
Kanamycin^§^	8	4 (10.8)		33 (89.2)	3 (15.8)		16 (84.2)	7 (12.5)		49 (87.5)
Mupirocin^§^	1	0 (0)		37 (100)	0 (0)		19 (100)	0 (0)		56 (100)

*R*, resistant; *I*, susceptible, increased exposure; and *S*, susceptible. ^†^The susceptibility of 23 isolates could not be determined, because their minimal inhibitory concentration (MIC) fell below the lowest concentration measured (0.25 mg/L). ^‡^MIC values evaluated according to the Clinical and Laboratory Standards Institute (CLSI) criteria. ^§^Epidemiological cut-off (ECOFF) values were used to determine wild-type (WT) and non-wild-type isolates.

**Table 2:** Antimicrobial resistance determinants in 56 swine-related Livestock-Associated Methicillin-Rresistant* Staphylococcus aureus* isolates. Resistance genes and mutations are listed as identified using ResFinder. The occurrence rates are indicated in parentheses.

Antimicrobial classes	Resistance genes (%)
Beta-lactams	*blaZ* (100); *mecA* (100)
Tetracyclines	*tet*(M) (98.2); *tet*(K) (96.4); *tet*(L) (5.4)
Phenicols	*fexA* (28.6)
Aminoglycosides	*aac(6′)-aph(2″)* (12.5); *aadD* (5.4); *ant(9)-Ia* (50.0)
Streptomycin	*ant(6)-Ia* (5.4); *str* (19.6)
Trimethoprim	*dfrG* (44.6); *dfrK* (12.5)
Fusidic acid	*fus*(C) (1.8)
Lincosamides	*lnu*(A) (5.4); *lnu*(B) (48.2)
Lincosamides, pleuromutilins, streptogramin A	***lsa*(E)** (48.2); ***vga*(A)**_**LC**_ (10.7); ***vga*(A)**_**V**_ (8.9); ***vga*(E)** (3.6)
Macrolides, lincosamides, streptogramin B	***erm*(A)** (5.4); ***erm*(B)** (10.7); ***erm*(C)** (26.8)
All phenicols, lincosamides, oxazolidinones, pleuromutilins, streptogramin A	***cfr*** (1.8)
	Gene mutations and corresponding amino acid change
Fluoroquinolones	*gyrA* [S84L] (33.9) *grlA* [S80F] or *grlA* [S80Y] (48.2)

Genes with bold characters are multidrug resistance genes, that is, they code resistance to at least three antimicrobial classes.

## Data Availability

The data that support the findings of this study are available in the Supporting Information files of this article. Sequencing data of the Hungarian LA-MRSA strains have been submitted to the NCBI Sequence Read Archive database under the BioProject accession numbers PRJNA901421 and PRJNA893357. Previously reported sequencing data of Danish LA-MRSA strains were used to support this study and are available at the European Nucleotide Archive under BioProject accession number PRJEB25608. This prior study (and dataset) is cited at relevant places within the text as reference [23].

## Supplementary Materials

Supplementary Materials S1: Bioinformatic pipeline of the single nucleotide polymorphism (SNP) analysis of methicillin-resistant* Staphylococcus aureus* strains. S2: Major characteristics of methicillin-resistant* Staphylococcus aureus* strains included in the study. S3: Discrepancies between antimicrobial resistance phenotype and genotype in 56 swine-related livestock-associated methicillin-resistant* Staphylococcus aureus* isolates. S4: Core genome multilocus sequence typing (cgMLST) and single nucleotide polymorphism (SNP) distance matrices of the livestock-associated methicillin-resistant *Staphylococcus aureus* isolates. S5: Relatedness of Hungarian and Danish methicillin-resistant* Staphylococcus aureus* clonal complex (CC) 398 isolates based on the core genome multilocus sequence typing (cgMLST) analysis.
