# Supplementary material for: High Prevalence of Livestock-Associated Methicillin-Resistant Staphylococcus aureus in Hungarian Pig Farms and Genomic Evidence for the Spillover of the Pathogen to Humans
Source: Transbound Emerg Dis. 2023 Feb 21;2023:5540019. doi: 10.1155/2023/5540019 (PMC12017024; doi:10.1155/2023/5540019)
Supplement: Supplementary Materials — S1: Bioinformatic pipeline of the single nucleotide polymorphism (SNP) analysis of methicillin-resistant Staphylococcus aureus strains. S2: Major characteristics of methicillin-resistant Staphylococcus aureus strains included in the study. S3: Discrepancies between antimicrobial resistance phenotype and genotype in 56 swine-related livestock-associated methicillin-resistant Staphylococcus aureus isolates. S4: Core genome multilocus sequence typing (cgMLST) and single nucleotide polymorphism (SNP) distance matrices of the livestock-associated methicillin-resistant Staphylococcus aureus isolates. S5: Relatedness of Hungarian and Danish methicillin-resistant Staphylococcus aureus clonal complex (CC) 398 isolates based on the core genome multilocus sequence typing (cgMLST) analysis. [file 5540019.f1.zip › Supporting Information S1 (2).docx]

**Supporting Information S1 |** *Bioinformatic pipeline of the single nucleotide polymorphism (SNP) analysis of methicillin-resistant* Staphylococcus aureus *strains*

Quality of raw sequencing reads was evaluated by FastQC v0.11.7 [1] and MultiQC [2]. Trimming of the reads was performed with TrimGalore v0.6.6 [3] with 30 as the quality threshold and 50 as the minimum read length.

Trimmed reads were aligned to the *S. aureus* reference sequence NC_007795.1 (downloaded at: 2022.10.25) with Bowtie2 v2.4.5 [4]. Duplicate sequences were removed with Picard v2.22.2 [5]. Pileups were constructed with Samtools v1.14 [6, 7] and SNP variant calling was performed with VarScan v2.4.4 [8], using the mpileup2snp command with the following parameters: --min-coverage 10, --min-avg-qual 20, --min-var-freq 0.90, --strand-filter 1, --output-vcf 1. Consensus genomes were constructed with bcftools v1.9 [6] and masked at positions where the depth of coverage was less than 10. Predicted recombination sites were masked with Gubbins v3.2.1 [9] from further analysis.

Phylogenetic analysis was performed in R environment [10]. Maximum likelihood phylogeny was computed with the ape v5.6.2 [11], phangorn v2.10.0 [12] and Biostrings v2.64.1 [13] R packages. GTR+G+I was selected as the optimal nucleotide substitution model based on the AIC results computed by the modelTest function of the phangorn package. 1000 iteration bootstrap analysis was performed on the trees to assess the reliability of the branches.

**References:**

1 Andrews S. (2012). FastQC: a quality control tool for high throughput sequence data. Available online at: <http://www.bioinformatics.babraham.ac.uk/projects/fastqc>

2 Ewels, P., Magnusson, M., Lundin, S., & Käller, M. (2016). MultiQC: summarize analysis results for multiple tools and samples in a single report. Bioinformatics, 32(19), 3047-3048.

3 Krueger, F. (2020). Trim Galore https://github.com/FelixKrueger/TrimGalore.

4 Langmead B, Salzberg S. Fast gapped-read alignment with Bowtie 2. Nature Methods. 2012, 9:357-359.

5 “Picard Toolkit.” 2019. Broad Institute, GitHub Repository. https://broadinstitute.github.io/picard/; Broad Institute

6 Danecek P, Bonfield JK, Liddle J, Marshall J, Ohan V, Pollard MO, Whitwham A, Keane T, McCarthy SA, Davies RM, Li H, Twelve years of SAMtools and BCFtools, GigaScience (2021) 10(2) giab008 [33590861]

7 Li H, A statistical framework for SNP calling, mutation discovery, association mapping and population genetical parameter estimation from sequencing data, Bioinformatics (2011) 27(21) 2987-93. [21903627]

8 Koboldt, D., Zhang, Q., Larson, D., Shen, D., McLellan, M., Lin, L., Miller, C., Mardis, E., Ding, L., & Wilson, R. (2012). VarScan 2: Somatic mutation and copy number alteration discovery in cancer by exome sequencing Genome Research DOI: 10.1101/gr.129684.111

9 Croucher, N. J., Page, A. J., Connor, T. R., Delaney, A. J., Keane, J. A., Bentley, S. D., ... & Harris, S. R. (2015). Rapid phylogenetic analysis of large samples of recombinant bacterial whole genome sequences using Gubbins. Nucleic acids research, 43(3), e15-e15.

10 R Core Team (2021). R: A language and environment for statistical computing. R Foundation for Statistical Computing, Vienna, Austria. URL https://www.R-project.org/.

11 Paradis E, Schliep K (2019). “ape 5.0: an environment for modern phylogenetics and evolutionary analyses in R.” Bioinformatics, 35, 526-528.

12 Schliep K.P. 2011. phangorn: phylogenetic analysis in R. Bioinformatics, 27(4) 592-593

13 H. Pagès, P. Aboyoun, R. Gentleman and S. DebRoy (2022). Biostrings: Efficient manipulation of biological strings. R package version 2.64.1. <https://bioconductor.org/packages/Biostrings>
