# Supplementary material for: High Prevalence of Livestock-Associated Methicillin-Resistant Staphylococcus aureus in Hungarian Pig Farms and Genomic Evidence for the Spillover of the Pathogen to Humans
Source: Transbound Emerg Dis. 2023 Feb 21;2023:5540019. doi: 10.1155/2023/5540019 (PMC12017024; doi:10.1155/2023/5540019)
Supplement: Supplementary Materials — S1: Bioinformatic pipeline of the single nucleotide polymorphism (SNP) analysis of methicillin-resistant Staphylococcus aureus strains. S2: Major characteristics of methicillin-resistant Staphylococcus aureus strains included in the study. S3: Discrepancies between antimicrobial resistance phenotype and genotype in 56 swine-related livestock-associated methicillin-resistant Staphylococcus aureus isolates. S4: Core genome multilocus sequence typing (cgMLST) and single nucleotide polymorphism (SNP) distance matrices of the livestock-associated methicillin-resistant Staphylococcus aureus isolates. S5: Relatedness of Hungarian and Danish methicillin-resistant Staphylococcus aureus clonal complex (CC) 398 isolates based on the core genome multilocus sequence typing (cgMLST) analysis. [file 5540019.f1.zip › Supporting Information S3 (1).docx]

**Supporting Information S3 |** Discrepancies between AMR phenotype and genotype in 56 swine-related livestock-associated methicillin-resistant Staphylococcus aureus (LA-MRSA) isolates.

| **Isolate** | **AMR phenotype** | **MIC value**  **(mg/L)** | **AMR genotype** |
| --- | --- | --- | --- |
| F37_E1 | CLI^S^ | ≤ 0.12 | *lnu*(A) |
| F06_E1, F07_E1, F08_E1, F12_H1, F13_E1, F20_E1, F31_E1, F40_E1, | CHL^R^ | 16 | ND |
| F25_H1, F30_H1 | KAN^S^ | ≤ 4 | *aadD* |
| F20_E2, F21_H1; F37_H1 | Q/D^I^ | 2 | *vga*(A)_V_ with *erm*(C), or *vga*(E) with *erm*(A) |
| F03_E1, F05_E1, F08_E2, F09_E1, F11_E1, F11_H1, F14_E1, F25_H1, F28_E1, F28_E2, F28_H2, F30_E1, F32_H1, F34_E1 | Q/D^R^ | ≥ 4 | *lsa*(E) |
| F07_E1; F12_H1 | CIP^I^ | 1 | *grlA* [S80F or S80Y] |

Resistance genes and mutations as identified by the ResFinder tool. CLI: clindamycin; CHL: chloramphenicol; KAN: kanamycin; Q/D: quinupristin/dalfopristin; CIP: ciprofloxacin; ND: no corresponding genetic trait detected.
