# Supplementary material for: High Prevalence of Livestock-Associated Methicillin-Resistant Staphylococcus aureus in Hungarian Pig Farms and Genomic Evidence for the Spillover of the Pathogen to Humans
Source: Transbound Emerg Dis. 2023 Feb 21;2023:5540019. doi: 10.1155/2023/5540019 (PMC12017024; doi:10.1155/2023/5540019)
Supplement: Supplementary Materials — S1: Bioinformatic pipeline of the single nucleotide polymorphism (SNP) analysis of methicillin-resistant Staphylococcus aureus strains. S2: Major characteristics of methicillin-resistant Staphylococcus aureus strains included in the study. S3: Discrepancies between antimicrobial resistance phenotype and genotype in 56 swine-related livestock-associated methicillin-resistant Staphylococcus aureus isolates. S4: Core genome multilocus sequence typing (cgMLST) and single nucleotide polymorphism (SNP) distance matrices of the livestock-associated methicillin-resistant Staphylococcus aureus isolates. S5: Relatedness of Hungarian and Danish methicillin-resistant Staphylococcus aureus clonal complex (CC) 398 isolates based on the core genome multilocus sequence typing (cgMLST) analysis. [file 5540019.f1.zip › Supporting Information S5 (2).docx]

**Supporting Information S5 |** *Relatedness of Hungarian and Danish methicillin-resistant* Staphylococcus aureus *clonal complex (CC) 398 isolates based on the core genome multilocus sequence typing (cgMLST) analysis.* Danish isolates belong to lineages that were identified in Denmark labelled as follows: Lineage 1 (green circle), Lineage 2 (blue rectangle), Lineage 3 (red triangle). The identified genetic clusters of Hungarian isolates (C1-5) are also highlighted. The tree scale bar indicates 10 allelic difference.

**C2A**

**C1A**

**C4A**

**C5A**

**C3A**


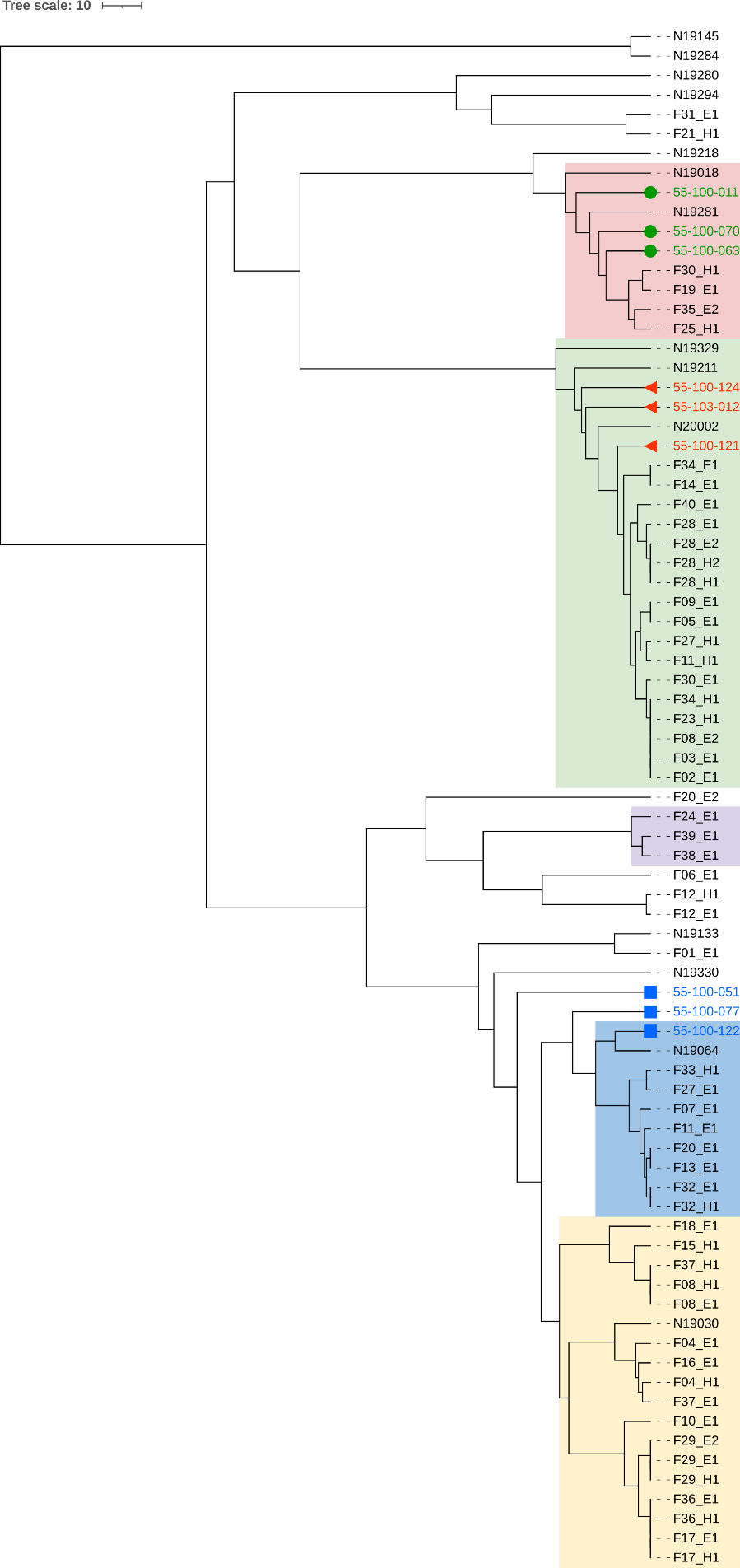


> 200

Group 1

Group 2
